# Color preferences affect learning in zebrafish, *Danio rerio*

**DOI:** 10.1038/s41598-019-51145-5

**Published:** 2019-10-10

**Authors:** Tamal Roy, Piyumika S. Suriyampola, Jennifer Flores, Melissa López, Collin Hickey, Anuradha Bhat, Emília P. Martins

**Affiliations:** 1 School of Life Sciences, Arizona State University, 427 E Tyler Mall, Tempe, AZ 85287 USA; 2 Department of Biological Sciences, Indian Institute of Science Education and Research Kolkata, Mohanpur, 741246 West Bengal India

**Keywords:** Behavioural ecology, Animal behaviour

## Abstract

Animals may exhibit preference for colors that match their environment or the resources in the environment. These preferences may impact ability to learn associations with these colors and revert the associations when the reward contingency is modified. We used zebrafish *Danio rerio* from four populations to test if color preferences impact associative and reversal learning ability. First, we tested if preference for blue or green impact associative ability. We subjected individual fish through eight trials to associate a social stimulus with blue or green. Next, we tested if preference for red or green impact associative reversal learning ability. We trained fish in groups of three to associate a social stimulus with red or green over three trials, and reversed the reward contingency during the following session. Results showed that zebrafish preferred green over blue and domesticated fish chose green more than blue when there was a reward attached. Zebrafish also preferred red over green. Fish from one wild population learned with both colors and reversed learning only from green to red and not vice-versa. Fish from another population showed an overwhelming preference for red irrespective of what was rewarded. Domesticated fish did not show reversal learning ability.

## Introduction

Color preferences may impact learning, and the extent of this impact may depend on the type of learning as well as on the developmental and evolutionary history of the animals. Animals are often better able to perceive colors that contrast with their environments^1–3^, and such sensory abilities and consequent preferences depend on both evolutionary history^4^ and individual experience^5^. The impact of color preferences on learning may also depend on the specific type of learning. Animals differ in their cognitive flexibility, with some being more limited than others in their abilities to reverse learning^6,7^. We might expect species or populations that have evolved strong color preferences also to be more limited in their ability to reverse learning^8^. Here, we ask whether zebrafish, *Danio rerio*, that evolved and developed in different physical environments have different color preferences, and whether these preferences impact their associative and reversal learning abilities.

Animals often prefer colors that are either most represented in the environment^9^ or contrast with the background^10^. For example, the parasitic wasp, *Venturia canescens*, prefers yellow, which is the most abundant color among natural flowers in temperate regions^11^ while tropical Asian birds prefer red and black, which are the most commonly encountered forest fruit colors^12^. Great bowerbirds, *Chlamydera nuchalis*, prefer colors that contrast with their own plumage and the visual backgrounds adjacent to the bower^13^ while bumblebees, *Bombus impatiens*, prefer blue flowers that stand out in a complex background^14^. While a tendency to approach the most common colors may help animals find abundant food, preference for a color that contrasts with the background may help animals to assess mate quality or to locate less common resources^15^. Preference for colors based on higher conspicuousness or contrast may enable animals to easily learn associations with them.

Associative learning involves forming associations between a stimulus and resources resulting in a change in behavior over time^16^. The ability to learn associations with a color stimulus helps animals identify food (moth *Venturia canescens*^11^), locate mates (parasitoid wasps *Nasonia vitripennis*^16^) and hosts (parasitoid flies *Exorista sorbillans*^17^), and avoid toxic prey (European robins *Erithacus rubecula*^18^) and predators^19^. Moreover, the ability to modify a previously learned experience and develop a new one allows animals to cope with complex or predictable but rapidly changing environments^20^. This reversal learning has been well documented in several species like guppies *Poecilia reticulata*^20^, Pinyon jays *Gymnorhinus cyanocephalus*, scrub jays *Aphelocoma californica* and nutcrackers *Nucifraga columbiana*^21^, jumping spiders *Marpissa muscosa*^22^. However, animals may differ in associative and reversal learning tendencies. Animals showing strong associative learning ability may or may not be successful in reversing learning when the reward contingencies are reversed. For example, while pheasant chicks *Phasianus colchicus* learned to associate blue and green with food rewards and reversed learning when reward contingencies were flipped^23^, adult Florida scrub jays *Aphelocoma coerulescens* that learned to associate blue or green with food reward failed in reversal when the reward contingencies changed^24^. Modifying learned associations with preferred colors might not be easy when compared to making initial associations with the same^25^. Here, we test whether populations simultaneously differ in associative and reversal learning based on the colors they preferred.

Population differences in learning abilities are linked to environmental complexity and variability^7^. For example, populations may differ in ability to learn color associations with rewards (bumblebees *Bombus terrestris*^26^, zebrafish^27^) and these differences may be related to the heterogeneity of the environments^28^. Fluctuating environmental conditions would require animals to unlearn previously learned associations and develop new ones^6^. Since populations may face different levels of environmental fluctuations^29^, the tendency to reverse learning may be expected to vary across populations. Though inter-population studies on associative learning are common, variation in reversal learning across populations has received less attention (see review by^6^).

Zebrafish is an important vertebrate model system used frequently in cognitive behavioral studies^30,31^ with most studies utilizing color based discrimination for associative and reversal training. Most studies on associative learning in zebrafish have used laboratory strains (but see^27^) that develop and evolve in bare and homogenous environments. Wild zebrafish originate from a diverse range of habitats that differ in complexity of physical environments and water clarity^32–35^, and these differences in the environments play a significant role in shaping associative learning and memory in juveniles^36^ and adults^27,34^. While some studies on lab reared strains showed that zebrafish were attracted to shorter wavelength colors like blue and green and avoided longer wavelength colors like red and yellow^37,38^, other studies demonstrated that zebrafish strongly avoided blue but were attracted to green, red and yellow colors^39^. Here we used three wild-caught and one domesticated populations of zebrafish to test how differences in preference between blue and green, and red and green impact learning. Blue and green are colors of lower opponency and green matched the color of the environment while red and green are colors of higher opponency and red matched the color of the food^39,40^.

We first tested for population differences in color preference, and then tested for differences in associative and reversal learning. In our first experiment, we focused on simple associative learning with a blue or green stimulus, and asked whether zebrafish from different populations differed in both their preferences for these colors and their ability to learn an appetitive task using these stimuli. Next, we asked about reversal learning with a red or green stimulus, again starting with a test of population differences in color preferences.

## Results

In the initial color preference tests, wild-caught zebrafish in our study entered a green door rather than a blue door first, whether those fish were from populations A (Green: 69%, Blue: 31%; χ^2^_1_ = 4.5, n = 32, p = 0.03), B (Green: 69%, Blue: 31%; χ^2^_1_ = 3.8, n = 26, p = 0.05) or C (Green: 69%, Blue: 31%; χ^2^_1_ = 3.8, n = 25, p = 0.05). Although most zebrafish from the domesticated population D also entered the green door first, this preference was not statistically significant (Green: 60%, Blue: 40%; χ^2^_1_ = 1.0, n = 25, p = 0.32).

**Figure 1:**
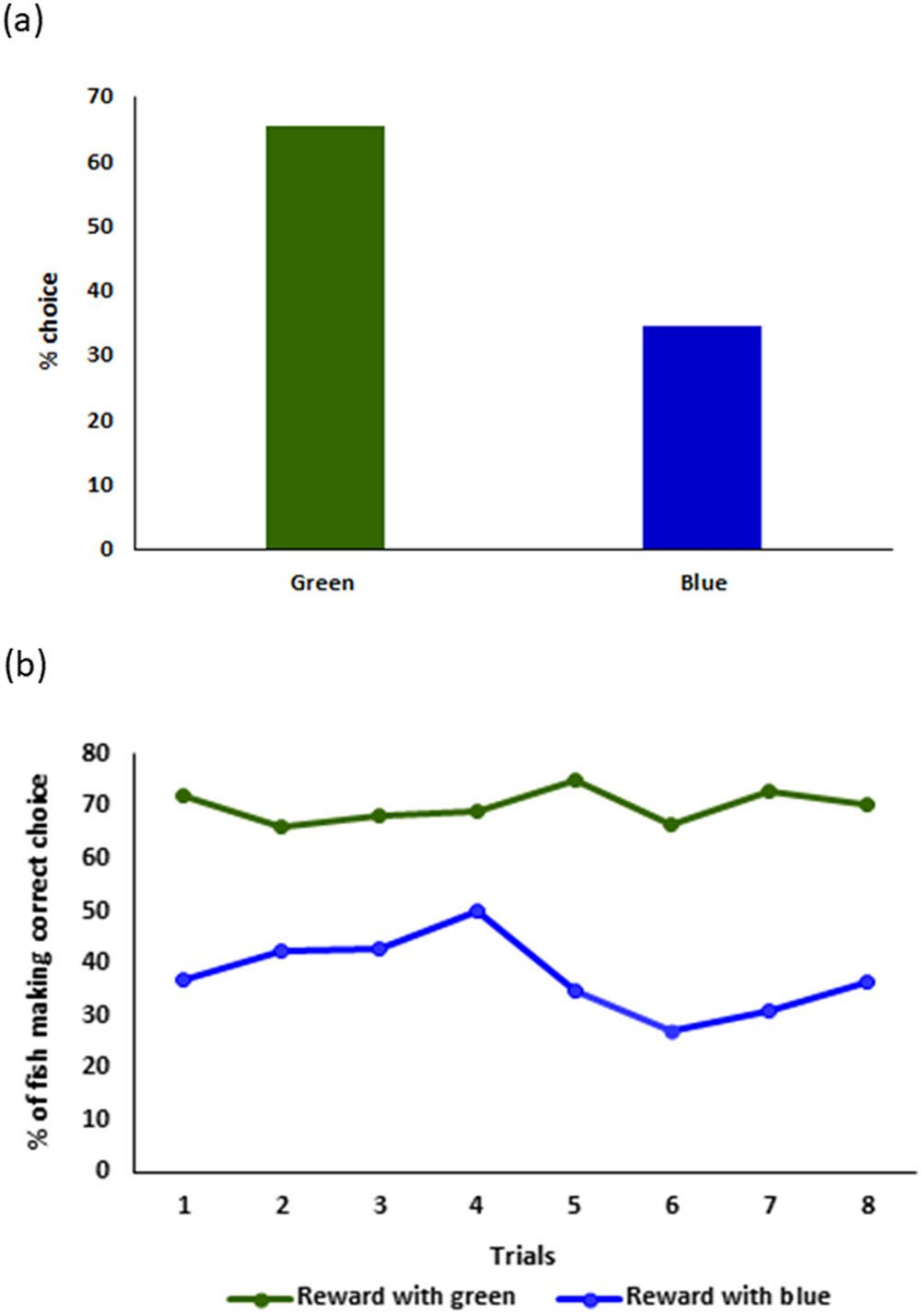
(**a**) Fish entered the green door more often (66%) than the blue door (34%) during associative sessions but the difference was not significant. (**b**) Percent of individuals making the choice of the door leading to social stimulus across trials during the green rewarded, blue unrewarded (represented with green line) and blue rewarded, green unrewarded (represented with blue line) training paradigms. Though higher percent of fish consistently chose green when the stimulus was at its side, there was no improvement in performance across trials.

Although zebrafish also entered the green door in 66% of our associative learning sessions (Fig. 1a), we did not find a significant main effect of reward color (z = 1.0, p = 0.33) or a significant interaction between reward color and population identity (p > 0.4 in all cases) in our repeated-measures logistic regression. We also did not find a significant effect of session (z = 1.1, p = 0.26) suggesting that zebrafish did not learn an association between the color of the door and the social reward after eight sessions (Fig. 1b). The only significant main effect, though marginal, in our logistic regression showed that fish from the domesticated population, D, differed, choosing the green door more often across eight sessions (70%; z = 2.0, p = 0.048) than did the wild-caught fish (A: 67%, B: 59%, C: 67%). We found no other significant differences between fish from different populations (p > 0.3).

**Figure 2:**
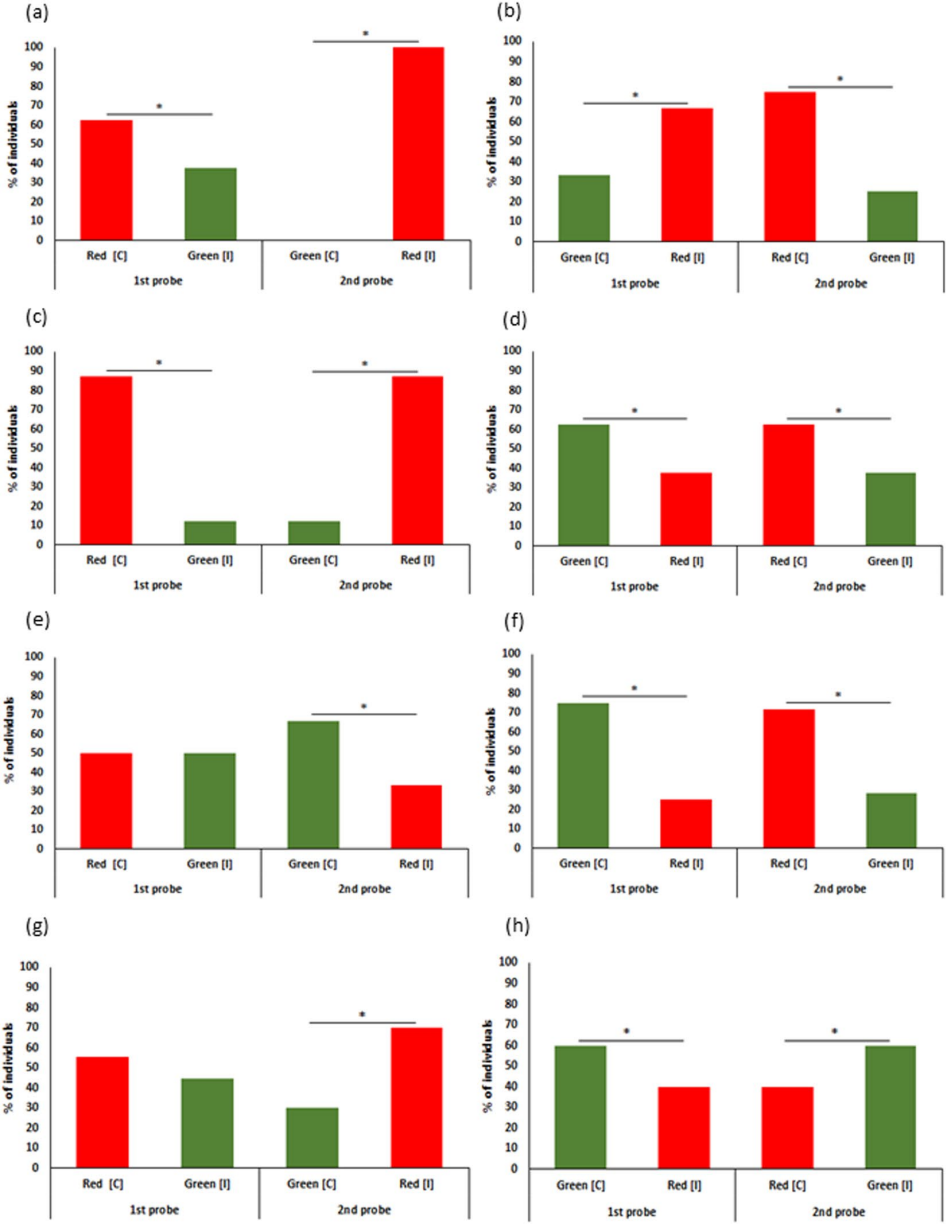
Results of red to green (**a**,**c**,**e** and **g**), and green to red (**b**,**d**,**f** and **h**) reversal learning assays of the four populations. The bars represent percent of individuals choosing red and green during 1^st^ and 2^nd^ probes (C = correct color, I = incorrect color). Significance is indicated by (*). (**a**) Significantly higher percent of fish from population C chose to enter the red door during both the 1^st^ and 2^nd^ probes. (**b**) Significantly higher percent of fish from population C chose to enter the red door during both the 1^st^ and 2^nd^ probes. (**c**) Significantly higher percent of fish from population A chose to enter the red door during both the 1^st^ and 2^nd^ probes. (**d**) Significantly higher percent of fish from population A chose to enter the green door during the 1^st^ probe and red door during the 2^nd^ probe. (**e**) Both red and green doors chosen equally by fish from population B during the 1^st^ probe while significantly higher percent of fish chose to enter the green door during the 2^nd^ probe. (**f**) Significantly higher percent of fish from population B chose to enter the green door during the 1^st^ probe and red door during the 2^nd^ probe. (**g**) Both red and green doors chosen equally by fish from population D during the 1^st^ probe while significantly higher percent of fish chose to enter the red door during both the 2^nd^ probe. (**h**) Significantly higher percent of fish from population D chose to enter the green door during both 1^st^ and 2^nd^ probes.

In the second experiment, zebrafish entered a red door first rather than a green door, whether the fish were from populations A (Red: 80%, Green: 20%; χ^2^_1_ = 7.2, n = 26, p = 0.01), B (Red: 75%, Green: 25%; χ^2^_1_ = 5.5, n = 21, p = 0.02), C (Red: 79%, Green: 21%; χ^2^_1_ = 6.4, n = 23, p = 0.01) or D (Red: 73%, Green: 27%; χ^2^_1_ = 6.5, n = 31, p = 0.01). We found that the preference for red over green impacted the choices made by fish from populations A and C during the probe trials after associative and reversal training. Using our repeated-measures logistic regression, we only found that fish from population C differed significantly from the other populations (population C main effect: z = 2.9, p < 0.01), and that there was a marginally non-significant effect of reward color (interaction between reward color and population C: z = −1.8, p = 0.06). Taking a closer look, we found that a significantly higher percent of C fish entered the red door during the first probe (associative learning) irrespective of whether the reward was associated with red (Red: 62%, χ^2^_1_ = 6.2, p = 0.01) or green (Red: 67%, χ^2^_1_ = 11.1, p < 0.01) (Fig. 2a,b) indicating strong preference for red. Again, a significantly higher percent of fish entered the red door during the second probe (reversal learning) when the reward was reversed from red to green (Red: 100%, χ^2^_1_ = 100.0, p < 0.01), and from green to red (Red: 75%, χ^2^_1_ = 25.0, p < 0.01) (Fig. 2a,b) indicating an overarching red preference. This suggests that a strong preference for the red color in population C constrained learning during associative and reversal sessions. No other interaction or main effects were statistically significant (p > 0.05), including the main effect for type of learning (associative or reversal, z = 1.4, p = 0.15).

Fish from population A did not differ significantly from the other populations with respect to the choice made during the probe trial in our logistic regression (z = 1.8, p = 0.07). Taking a closer look, a significantly higher percentage of fish from population A entered the red door (Red: 87%, χ^2^_1_ = 56.2, p < 0.01) when the reward was with red, and the green door (Green: 62%, χ^2^_1_ = 6.2, p = 0.01) when the reward was with green during the first probe (Fig. 2c,d) indicating associative learning with both colors. However, during the second probe (reversal learning), a significantly higher percentage of fish entered the red door irrespective of whether the contingency was reversed from green to red (Red: 87%, χ^2^_1_ = 56.2, p < 0.01) or red to green (Red: 62%, χ^2^_1_ = 6.2, p = 0.01) (Fig. 2c,d), indicating reversal only to red. This suggests that preference for red in fish from population A prevented reversal but not associative learning in these fish. Fish from populations B (z = −1.5, p = 0.13) (Fig. 2e,f) and D (z = 1.2, p = 0.22) did not differ significantly on the choice made during the probe trials (Fig. 2g,h).

## Discussion

Our results emphasize the importance of recent, developmental, and evolutionary history, demonstrating that zebrafish color preferences can impact learning but that the magnitude and type of impact depends on source population, as well as on the type of learning. In our blue-green discrimination task, whether there was a significant preference for green and whether green was chosen more than blue across eight sessions depended on population. In the red-green discrimination reversal task, we found that zebrafish preferred red over green. Fish from one population did not learn associations with a green color at all, and fish from a second population learned those associations only when they were presented first, and not when presented as a reversal learning task.

Color preferences may reflect both the environment in which fish evolve and develop^41^, and changes in visual sensitivity that may also vary with the spectral quality of the ambient light^42,43^. We found that zebrafish from the three wild populations entered a green door more than the blue door, but the domesticated population D entered a green door marginally more during associative sessions. Zebrafish may approach green more readily than blue because it is a familiar color in the natural habitat^40^. The ambient color of the tanks of our fish was blue but we did not find a positive effect of this on color preferences in wild fish. Some previous studies on domesticated strains have suggested that zebrafish avoid blue^39,40^ although others have found a preference for blue^37,44,45^. Our study confirms that domesticated zebrafish may have different preferences for green over blue than do wild zebrafish. Since we found We found zebrafish from all four populations to enter a red door more often than the green door. Previous studies showed that domesticated zebrafish show no preference for red over green in a two choice discrimination paradigm^39^ but preferred red over other colors when tested in a foraging context^40^. Here we show that in an exploratory context, wild and domesticated zebrafish approached red more readily than they did green. Red may be easier to detect in an aquatic environment^46–49^, and is likely a signal of food items such as zooplankton and microcrustaceans that contain dietary carotenoids^50^. Red is also the principal color of the artificial pellet food that is fed to the fish. Largemouth bass can more readily associate meaning to colors when presented with two colors of higher opponency like red and green^51^. Our results agree with these findings and also demonstrate that colors of longer wavelength are often more preferred than the shorter wavelength colors.

While the preference for green over blue marginally impacted behavioral decisions in the blue-green discrimination assay, preference for red over green impacted associative learning in one, and reversal learning in two populations of wild zebrafish in the red-green discrimination assay. We found little evidence of learning in the blue-green assay which contradicts previous studies where zebrafish have been shown to learn simple associative tasks over successive trials. For example, Roy and Bhat^27^ showed that wild zebrafish can learn to associate colors with food when trained daily for eight days. The specific color and whether the task is appetitive may be important, since Vital and Martins trained zebrafish from both wild and domesticated strains to associate red with a food reward over three days^52^, and to associate blue with an aversive stimulus over three sessions on a single day^53^. The use of food instead of social reward could have led to higher initial responses but the fish would have been satiated quickly since the trials were conducted in one day. This is because zebrafish is a small fish that does not need to forage at regular short intervals. Our current study suggests that condensing training into a single day to avoid satiation^54^ might have impeded learning. Further, repeated training of single individuals might have increased stress and impacted performance since zebrafish is a highly social species. We observed learning in the red-green discrimination assay where fish were trained as groups of three across three sessions. Here, a strong preference for red constrained associative learning in fish from population C (Fig. 2a,b) while fish from population A learned the association equally well with red and green (Fig. 2c,d). The association with red was stronger than with green in population A. The two other populations B (Fig. 2e,f) and D (Fig. 2g,h) showed associative learning only with green. A recent study on largemouth bass showed that the red color was easy to identify when fish were trained to red^51^, since bass are particularly attracted to the red color^47,48^. Our findings from population A are in agreement with this, and other studies on zebrafish where wild fish easily attached meaning to red^27,55^. Since these fish also learned with green, preference for red does not seem to impact associative learning ability. Fish from population C had the strongest preference for red among all populations since they always went to red regardless of whether a stimulus was attached to it. This is similar to previous findings in a parasitic wasp *Venturia canescens* where the choice for the innately preferred color yellow was not modified by associative learning^11^. The preference for red in population A was modified by associative learning as the fish also learned with green.

Our results demonstrate that preference for colors impact reversal learning ability in animals. While fish from population A learned an association with both red and green, they reversed learning only from green to red and not from red to green (Fig. 2c,d). Therefore, a strong preference for red prevented fish from unlearning a previously learned association with red and learning a new one. This contrasts the findings of Mitchem, *et al*.^51^ where largemouth bass, after being previously trained to red, avoided red when trained to other colors. On the other hand, fish from population C demonstrated an overarching preference for red irrespective of what was rewarded (Fig. 2a,b). This finding about constraint on learning due to strong preferences, and inability to revert a learned association with a more preferred to a less preferred color in wild fish is significant since earlier studies on color reversal learning in zebrafish^56,57^ and other species (guppies^20^, birds^21^, bumblebees^58^) have not regarded the influence of color preferences on reversal ability. The fish from these populations belong to habitats with high turbidity (Table 1). Visual system of aquatic animals evolve and develop based on the spectral characteristics of the water they inhabit^41,59^. Bluefin killifish *Lucania goodei* from swamp habitats are more sensitive to longer wavelengths^60^, and individuals raised under turbid conditions show high pecking preference for red objects^41^. Zebrafish from turbid habitats could, similarly, prefer red. A constraint on learning^61^ due to the strong preference for red could enable them in long range detection of targets. Population B reversed learning from less preferred color green to more preferred color red which meant that though they did not initially learn with red, they reversed learning to red easily (Fig. 2e,f). Population D did not show reversal learning ability at all (Fig. 2g,h) and this contradicts previous studies that reported reversal learning abilities in lab reared strains of zebrafish^56,57^. These results support our prediction that wild fish will demonstrate reversal learning ability while the domesticated fish will not. This difference could be because the wild fish needed to be more flexible with learning to cope with unpredictable environmental changes while the domesticated population may have lost the ability to cope with these conditions. Further studies with more populations would help us to understand how cognitive reversal ability may vary with environmental fluctuations. Previous studies have looked into improvement in reversal learning over subsequent trials^20,58,62^. We observed reversal in a single probe trial, but the reversal tendency significantly differed based on the reversal paradigm employed which supports our prediction about the impact of color preferences on reversal learning in zebrafish.

**Table 1:** Mean values (SE) of the habitat measures recorded at the three Indian sites A, B and C.

	A	B	C
**At zebrafish groups**			
Flow rate (cm/s)	0.0 (0.00)	14.1 (1.09)	0.0 (0.00)
Vegetation cover (%)	5.0 (2.32)	49.1 (15.38)	32.47 (4.1)
Depth (cm)	12.5 (1.73)	10.4 (0.75)	28.9 (4.1)
**General measures**			
Temperature (°C)	25.6 (0.06)	26.5 (0.17)	25.7 (0.4)
pH	7.4 (0.12)	7.5 (0.03)	6.1 (0.2)
Total dissolved solids (ppt)	0.3 (0.00)	0.2 (0.00)	38.33 (0.5)
Apparent color (Pt-Co)	62.3 (18.22)	33.7 (5.90)	259.33 (3.9)
Ammonia (ppm)	0.3 (0.00)	0.8 (0.08)	0.05 (0.00)
Substrate	Mud	Silt	Mud
Elevation (m)	12.2	41.4	28

The measures for populations A and B are from Suriyampola *et al*.^35^, and the measures for population C were taken in the similar way.

## Conclusion

Here, we find that fish from different source populations differ in the magnitude of their preferences and the impact of those preferences on learning, suggesting that developmental and evolutionary history may play important roles. Preferred colors like red and green are easily learned and attract higher receiver responses. Overall, red is the most strongly preferred color and associations with red are often easily learned, but not unlearned. These behavioral responses towards red might enable long range detection of food in fish as it is the case in other species^46,63^. In our study, zebrafish approached the longer wavelength color (green or red) more readily when given a choice between consecutive (blue-green) or distant (green-red) colors in the visible spectrum. Colors of lower opponency (blue-green) did not elicit distinct differences in learning while the colors of higher opponency (green-red) did, which strongly indicates that selection of colors for two-choice discrimination tasks is crucial. Importantly, the strength of the color preference determined if fish were able to revert a previously learned association. Fish differed in reversal ability based on the reward paradigm as well as the population origin. Zebrafish is an important vertebrate model in cognitive studies where it is regularly trained on color-based discrimination tasks for associative and reversal learning. Our results suggest a cautious choice of color cues for such studies because the results obtained could be strongly confounded by preferences.

## Methods

### Subjects and maintenance

We used zebrafish from three wild and one domesticated populations for this study. During the post-monsoon season in November 2017, we collected fish from three Indian populations: ‘A’, a stagnant agricultural drain with turbid water, emergent vegetation, and mud, dead plant matter and inorganic waste as substrate (also termed KB^32,34,36,64^ and SN^35^), ‘B’, a fast flowing stream with clear water, vegetated and non-vegetated patches, and silt as substrate (also termed FM^35^), and ‘C’, a slow-flowing canal with highly turbid water, submerged and emergent vegetation, and silt as substrate. The habitat measures recorded at ‘A’ and ‘B’ (as in Suriyampola *et al*.^35^) and ‘C’ are shown in Table 1. In addition, we used captive-bred zebrafish ‘D’ obtained from Rawlins Tropical Fish Farm in Lithia, Florida, United States. The captive-bred fish were maintained at regular hatchery conditions. We housed the fish in laboratory in bare 8.5 L tanks in Zebtec Active Blue Tecniplast system^®^ in mixed-sex groups of approximately 15 individuals and 14:10 h light: dark cycle conditions. Water in the housing tanks had temperature 28 ± 1 °C, pH 7.4 and 400 µS conductivity, and light intensity at the center of tanks ranged from 176–251 Lux. We fed the fish daily with commercial flake food (Tetramin Tropical). The fish populations were maintained in the laboratory for five months before being used for the experiments. We did not record the body size and sex of the fish used in the experiments since a recent study on wild-caught zebrafish from four populations showed no consistent effects of body size and sex on a spatial performance task^27^.

### Experimental setup

**Figure 3:**
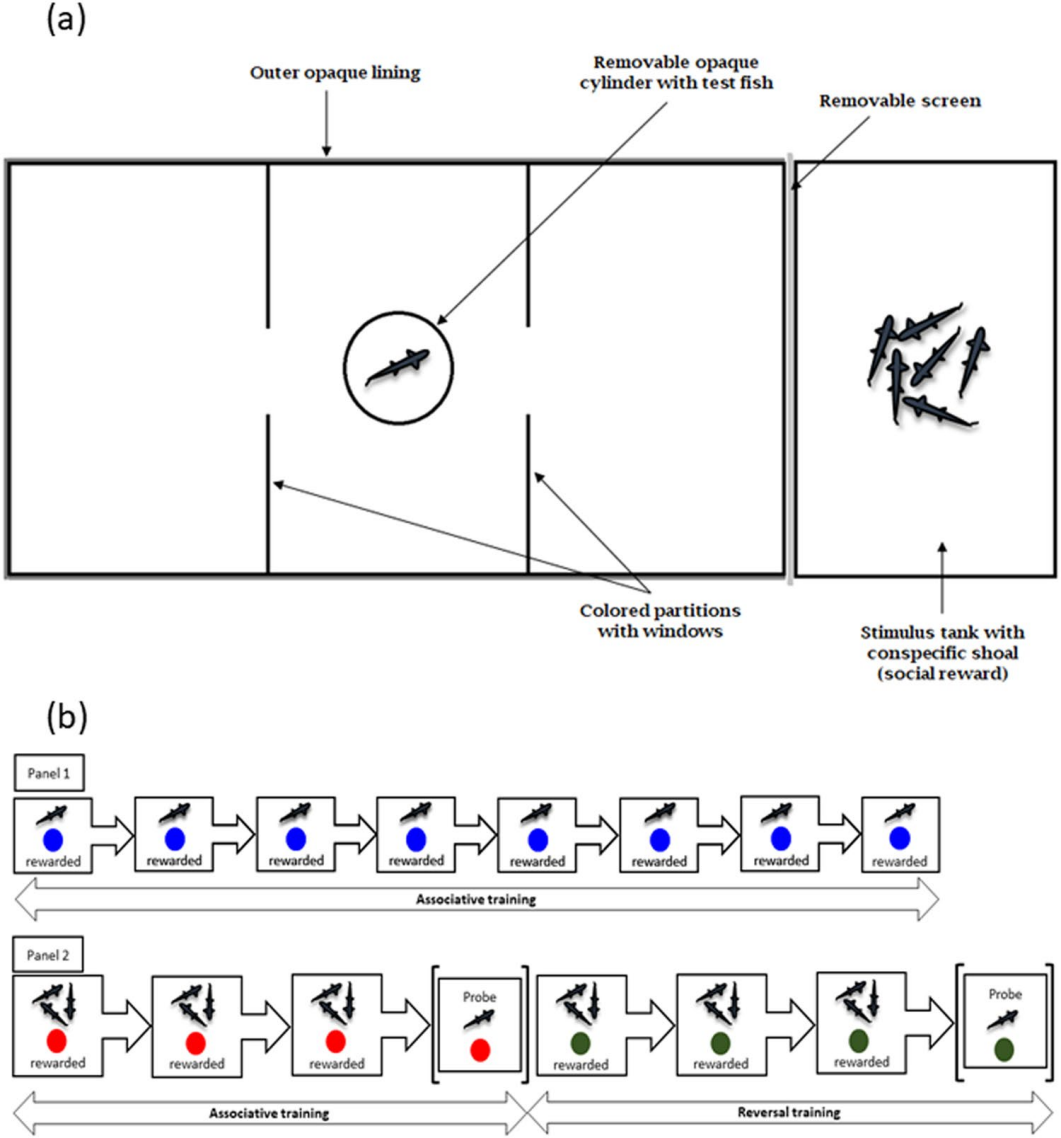
(**a**) Diagrammatic representation of the experimental setup (top-view). (**b**) Schematic representation of the experiments: Panel 1 shows testing associative ability of individual fish in a blue-green discrimination task through eight consecutive sessions where the reward is associated with blue. Similar protocol was followed for associative training with green. Panel 2 shows training of three fish through three sessions in a red to green reversal task followed by probe trial with a single individual. The first half shows test for associative ability where the reward is with red color and the second half shows test for reversal ability where the reward contingency is reversed to green color. The test for green to red reversal ability was conducted similarly.

We used a custom-made rectangular acrylic tank (28.8 cm × 14.6 cm × 12 cm) for testing the performance of adult zebrafish in a two-choice discrimination task (Fig. 3a). We divided the rectangular tank lengthwise into three equal-size compartments; the two flanking choice compartments and the central release compartment using removable colored opaque partitions (Fig. 3a). Each partition had a 2.54 cm × 2.54 cm window in the center that zebrafish could easily pass through. We covered the outside of the long sides of the arena with a white foam sheet to block visual access to external cues. The experiment room was lit by 48” retrofit LED lights (Revolution Lighting Technologies) and the light intensity in the middle of the room was 318.33 ± 0.3 Lux.

### Procedure

#### Two choice discrimination task using blue and green

We trained individual fish in a day over eight training sessions with a social reward to test for associative learning with blue vs. green stimuli (Fig. 3b). We used the first choice made by the fish during the very first session as a measure of color preference since the fish were then unaware of presence of any reward. We tested a total of 108 fish from four populations (A:32, B:26, C:25, D:25). Before beginning each training session, we transferred six zebrafish from the same population to a holding tank (14.6 cm × 12 cm × 9.6 cm) immediately adjacent to one end of the long arms of the test arena to serve as a social reward^65,66^. We did not use food reward like other studies^27,55^ so that the fish are not satiated over consecutive trials in a day. Since zebrafish is a gregarious species, we expected a higher response with a social stimulus compared to food reward. We paired half of the subject fish from each population with a social reward on the side marked by the green door, and the other half with a social reward near the blue door. We separated the social stimulus from the main test arena with a removable opaque screen (Fig. 3a) and allowed them to acclimate for 10 min. The other side of the test arena that did not have the social reward had a similar screen in place. We then placed a single subject zebrafish in an opaque cylinder in the center of the experimental arena. After a 1-min acclimation period, we removed the opaque cylinder by pulling a string from above, and began video-recording the fish using a Panasonic HC-V750 HD camcorder for a 10-min trial. After the subject fish entered the green or blue door nearest the stimulus tank for the first time, we removed the opaque screen so that they could see the social stimulus. At the end of each 10-min training session, we gently replaced the subject fish in the opaque cylinder in the center of the experimental arena and waited 10 min before starting with the next session. During this period, we moved the position of the doors, the stimulus tank and opaque screen randomly to prevent side bias. We repeated this process for a total of eight consecutive training sessions with each subject fish. From the video recordings, we scored the color of the door the fish entered first after release into the arena. We used the choice made by the fish for the first time as a measure of color preference, and the scores across eight sessions to test for population differences in simple associative learning. The fish were returned to the housing tanks and fed normally after the trials for the day were completed.

#### Two choice discrimination and reversal using red and green

Second, we used a group training paradigm^67^ with a social reward to test for population differences in reversal learning with red vs. green stimuli (Fig. 3b). Since we did not observe learning with single individuals possibly due to stress, we trained fish in groups in this experiment. We first tested for red-green color preference using a total of 101 zebrafish from four populations (A:26, B:21, C:23, D:31), using the same procedure as when testing for blue-green preference above. Next we measured reversal learning for a total of 68 subject fish (A:16, B:14, C:18, D:20). The blue-green assay, tests for red-green color preferences and the reversal learning assay were separated in time by two months to prevent habituation with the setup from affecting the results. The reversal learning trials for an individual subject were conducted in a single day. For each reversal learning trial, we began by choosing a subject and two companion fish (selected randomly) from each population such that the subject was easy to distinguish (e.g., slightly larger or smaller) from the other two. We used the same training arena as above with red and green doors, placing a clearly-visible social reward of six fish behind the red door for half of the subject fish and behind the green door for the remaining half. We then conducted a series of three training sessions, placing groups of three fish (one subject and two companions) into the central chamber of the arena, and allowing them to explore together for 30 min. After 30 min, we moved the subject and companion fish to a holding tank for 10 min before repeating for a total of three training sessions (Fig. 3b). After the third session, we removed the social reward and conducted a 10 min probe test by placing the subject fish alone into the test arena in an opaque cylinder and raising that cylinder remotely after a 1-min acclimation period. We recorded the fish behavior and scored the color of the door the subject fish entered first from the video-recordings. We conducted these initial trials in the morning, allowed the subject fish to rest with their two companions for 1 h and then ran a second set of training sessions and probe test in the afternoon to test reversal learning. In the afternoon session, we reversed the reward contingency for each fish by placing the social reward behind the door with the alternate color (Fig. 3b). After the trials for the day were completed, we returned the fish to the housing tanks and fed them normally.

### Ethical note

All procedures performed in these studies were in accordance with the guidelines and regulations of Institutional Animal Care and Use Committee (IACUC) of Arizona State University, USA. The experimental protocols were approved by IACUC of Arizona State University (ASU IACUC 17-1596).

### Statistical analysis

We used chi-square tests to evaluate color preference for zebrafish from each population separately as a difference in the proportion of fish entering blue/green or red/green doors first. For the blue-green discrimination sessions, we used a repeated-measures logistic regression to test whether reward Color, source Population and their interaction predicted the choice made by fish, while taking into account changes across the eight training sessions. For the reversal learning assay, we used a repeated measures logi stic regression to test whether reward Color, source Population and their interaction predicted the choice made by the fish during a probe test, while taking the learning type (associative or reversal) also into account. In both cases, we also conducted posthoc comparisons. We used the base functions and the lme4 package^68^ in R version 3.4.4 to conduct all analyses^69^. All data are provided in the Supplementary file.

## Supplementary information

Dataset 1, Dataset 2

## Data Availability

The data generated during this study is available as a Supplementary File.
